# Effect of One-Day Delaying CIDR Administration in 5-Day Cosynch Protocol in Dairy Heifers

**DOI:** 10.3390/ani11051402

**Published:** 2021-05-14

**Authors:** Sükrü Metin Pancarci, Örsan Güngör, Osman Harput, Oguz Calisici

**Affiliations:** 1 Faculty of Veterinary Medicine, Balıkesir University, Balıkesir 10145, Turkey; pancarci@balikesir.edu.tr; 2 Faculty of Veterinary Medicine, Burdur Mehmet Akif Ersoy University, Burdur 15030, Turkey; gungororsan@hotmail.com; 3 UNSPED Dairy Farm Incorporation, Karaman 70500, Turkey; osmanharput@gmail.com; 4 Clinic for Cattle, University of Veterinary Medicine Hannover (Foundation), 30173 Hannover, Germany

**Keywords:** pregnancy, TAI, Cosynch, progesterone, follicle development, dairy heifer

## Abstract

**Simple Summary:**

In modern dairy farms, the optimum age for first calving is 23 to 25 months, to achieve this goal heifers having to conceive at 15 months of age using fixed-time artificial insemination. Despite the difference of follicular dynamics between heifers and cows, the dairy cow based synchronization protocols need to be modified for dairy heifers. We investigated the effect of one-day delaying administration of intravaginal progesterone device (CIDR) at the beginning of 5-day Cosynch protocol. Pregnancy per AI (P/AI) was significantly declined in CIDR-4 (4-day CIDR application instead of 5 days) group as heifers’ age proceeded. We have shown that there was no benefit of one-day delay of CIDR administration in 5-day Cosynch protocol on P/AI. However, higher P/AI in 5-day CIDR synchronization in aged heifers could reveal the necessity of longer progesterone administration in Cosynch protocol for better fertility.

**Abstract:**

Effect of one-day delaying progesterone administration at the beginning of 5-day Cosynch protocol was investigated in Holstein heifers for the first artificial insemination (AI). Heifers received a synchronized ovulation and timed AI (TAI) with CIDR inserted on day (d) 0 (CIDR-5; *n* = 206) or d 1 (CIDR-4; *n* = 192). In both group, GnRH was administered on d 0 followed by a single PGF_2α_ injection and CIDR removal five days later from GnRH. On d 8, TAI and GnRH administration were concurrently conducted. Heifers detected in estrus up to 24 h prior to TAI were inseminated without GnRH administration. Rates of ovulation, accessory CL formation and new dominant follicle development following initial GnRH injection did not differ between groups. P/AI did not differ between CIDR-4 (44.3%, [85/192]) and CIDR-5 (51.9%, [107/206]) groups, respectively. Pregnancy per AI (P/AI) was significantly (*p* < 0.01) declined as heifers’ age (12–13, 14, 15, 16 and17–21 months) proceeded in CIDR-4 group (55.6%, 52.1%, 37.9%, 35.7%, 32.4%) compared to those in CIDR-5 group (60.0%, 50.0%, 53.9%, 51.5%, 46.2%) respectively. In conclusion, there is no benefit for delaying CIDR administration in 5-day Cosynch protocol in dairy heifers. However, higher P/AI in CIDR-5 group in older heifers can be considered for reproductive management.

## 1. Introduction

Economic calculations showed that the optimum age for first calving is 23 to 25 months, to achieve this goal heifers having to conceive at 15 months of age [1]. In this context, a study of Holstein and British Friesian heifers with the first calving ages between 21–42 months reported that heifers calving around two years old had shorter calving intervals in the following years than those conceived later [2]. Similarly, it was shown that increase in age at the first calving led to decrease in life time daily milk production and decrease in probability of calving for a subsequent time [2].

Follicular dynamics differ in heifers and cows [3,4]. Therefore, in heifers, the pregnancy results following fixed-time protocols adapted from cow protocols are not satisfying [5]. Sartori et al. [6] reported that dairy heifers had more follicular waves than cows although interovulatory intervals were similar between heifers and cows. Moreover, cows ovulating later than heifers following luteolysis.

Rabaglino et al. [7] developed a new Cosynch protocol for heifers. This ovulation synchronization protocol begins with an injection of GnRH and concurrent intravaginal insertion of CIDR (controlled internal drug release) device including progesterone (P4) on day 0. Five days after the GnRH injection, PGF2α is injected and CIDR is removed (day 5). Timed artificial insemination (TAI) along with GnRH injection were performed three days (72 h) after CIDR removal (day 8).

Lima et al. [8] demonstrated that LH release and ovulation rate were lower with regards to GnRH injection one day after CIDR insertion in dairy heifers with high P4 concentrations than those with low P4 concentrations. Similarly, Carvalho et al. [9] demonstrated that induction of low P4 environment without total luteolysis following a half dose PGF2α administration two days prior to initiation of Ovsynch protocol increased ovulation rate following initial GnRH administration in lactating dairy cows. In addition, Spencer et al. [10] reported that pregnancy per artificial insemination (P/AI) did not differ in 5-day CIDR Cosynch protocol with or without initial GnRH administration for resynchronization in lactating dairy cows. Futhermore, Palomares et al. [11] has suggested a Monday to Friday 4-day CIDR Cosynch protocol to simplify the weekly routine of reproductive management in dairy or beef heifers. In that study, CIDR were inserted in both groups at the initiation of Cosynch protocol; however, in 4-day group the removal of CIDR was one day earlier than that in 5-day group [11]. Similarly, in an attempt of shortening Cosynch protocol, it has been showed that heifers treated with a 4-day CIDR-Cosynch protocol had smaller follicular diameter following CIDR removal, approximately 6 h longer interval from CIDR removal to ovulation, higher estradiol-17β concentations at TAI and lower LH peak concentration compared to heifers in 5-day CIDR-Cosynch protocol [12]. On the other hand, there is no detailed study about an insertion of CIDR one day after initial GnRH application in 5-day CIDR Cosynch protocol in dairy or beef heifers.

Oguge et al. [13] reported that a single oral administration of medroxyprogesterone acetate (MPA) decreased the rate of ovulation and diminished the increase in plasma concentrations of P4 and LH following copulation in rabbits without any impact on LH release in response to GnRH. Moreover, tendencies for lower rate of accessory CL formation and distruption in follicular growth following concurrent administration of GnRH and melengestrol acetate (MGA) feeding in beef cows were reported [14].

Therefore, the objective of this study was to investigate the concurrent or separate administrations of GnRH and CIDR at the beginning of 5-day Cosynch protocol with a 4- or 5-day CIDR insertion on ovarian dynamics and P/AI in Holstein dairy heifers.

## 2. Materials and Methods

### 2.1. Animals and Treatment

This study was conducted in five commercial dairy farms in Turkey. Holstein dairy heifers were housed in dry lots with headlocks, and were fed with similar total mixed ration to provide the requirements for a Holstein heifer (including alfalfa hay, corn silage, wheat hay and concentrate with at least 14% crude protein) once or twice daily with ad libitum access to water. Clinical examination of heifers has been performed prior to initiation of the study. The clinical examination included control of body temperature, any sign of systemic disease, foot problems, and rectal examination to determine the ovarian structures and reproductive disorders. Following clinical examination, clinically healthy heifers (*n* = 410) were randomly allocated to one of two groups (CIDR-5 or CIDR-4) for their first AI; however, 398 heifers (mean and median of age: 15 months; 12–21 months in range) were completed the study. Each heifer was used only once in this study. In CIDR-5 group (*n* = 206), heifers were enrolled in Cosynch protocol, and GnRH (gonadorelin acetate; 100 µg; Acegon^®^, Zoetis Animal Health, Istanbul, Turkey) was administered concurrent with intravaginal CIDR (1.38 g progesterone, CIDR 1380^®^, Zoetis Animal Health, Istanbul, Turkey) insertion (day 0), followed by PGF_2α_ (dinoprost tromethamine; 25 mg; Dynolytic^®^, Zoetis Animal Health, Istanbul, Turkey) injection and CIDR removal five days later (day 5). Timed artificial insemination (TAI) along with GnRH injection were performed three days (72 h) after CIDR removal (day 8). In CIDR-4 group (*n* = 192), heifers were enrolled to the same Cosynch protocol; however, CIDR was inserted one day after the initiation of Cosynch protocol.

**Figure 1:**
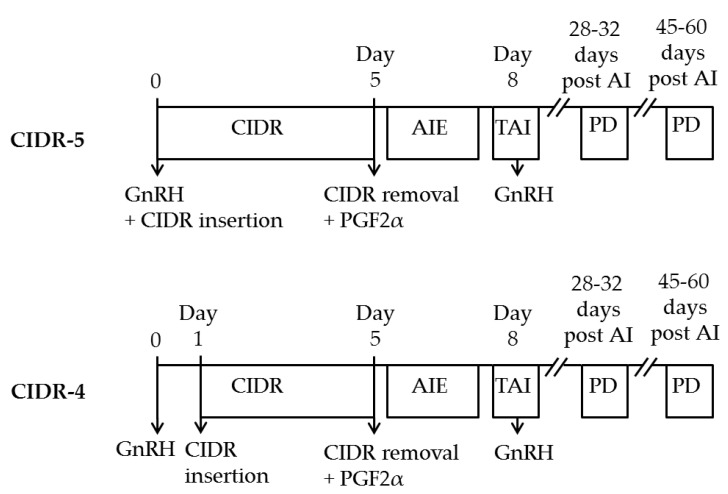
Diagrammatic representation of experimental design of treatments. CIDR, controlled internal drug release; TAI, timed artificial insemination; PD, Pregnancy diagnosis. Heifers at five locations were assigned randomly to receive a synchronization of ovulation and TAI. Both treatments consisted of a GnRH injection on day 0. CIDR inserted on day (d) 0 (CIDR-5; *n* = 206) or d 1 (CIDR-4; *n* = 192) of synchronization program. On d 8, TAI and GnRH administration were concurrently conducted. Heifers detected in estrus up to 24 h prior to TAI were inseminated without GnRH administration (AIE).

After the removal of CIDR, behavioral signs of estrus were visually observed at least two times a day (each observation for at least 30 min) in both groups in all farms. Heifers detected in standing estrus were inseminated (AIE) up to 24 h prior to scheduled TAI based on AM: PM and PM: AM rule. In case of AIE, heifers were not re-inseminated at TAI nor GnRH was administered following AIE. Conventional and sexed frozen-thawed semen were used based on farm management decisions. Sexed semen was not used in pre-synchronized heifers. Pregnancies were diagnosed with transrectal ultrasonography and palpation per rectum on days 28–32 and 45–60 days after insemination, respectively (Figure 1).

In subset of heifers (CIDR-4, *n* = 15; CIDR-5, *n* = 19) ovulation and formation of accessory corpus luteum (CL), development of new wave dominant follicle, original and accessory CL following initial GnRH administration were monitored with transrectal ultrasonography on days 0, 1, 2, 3, 4 and 5 of Cosynch protocol. Cosynch protocol was initiated at early diestrus, twelve days after the second PGF2α injection of presynchronization including PGF2α administrations 11 days apart. Twenty four heifers (CIDR-4, *n* = 9; CIDR-5, *n* = 15) having a visible CL (≥16 mm in average diameter of length and width) and a dominant follicle (DF ≥ 10 mm in diameter) at the beginning of 5-day Cosynch protocol completed this study. Heifers with early luteolysis during monitoring period were dropped from the study.

Including this subset of heifers, 78 heifers were pre-synchronized in one location. The remaining heifers (*n* = 320) were not pre-synchronized in all locations. Estrous detection and AI were not performed during and after presynchronization. Heifers were blocked by pre-synchronization or not pre-synchronization, and then randomly allocated to CIDR-5 or CIDR-4 groups as described above.

### 2.2. Statistical Analysis

In this study, all data were analyzed with statistical software program (SAS version 9.4 for Windows; SAS Institute, Cary, NC, USA). P/AI at 28–32 days, rates of TAI and AIE, and pregnancy loss between two pregnancy diagnosis were analyzed with logistic regression-stepwise elimination procedure of SAS. Whereas, rates of ovulation, accessory CL formation and development of new dominant follicle following initial GnRH injection were analyzed with Chi-Square analysis procedure of SAS. Heifers were retrospectively classified into five groups based on ages (months) at the beginning of Cosynch protocol as follows: 12–13, 14, 15, 16 and 17–21 months. Statistical model for P/AI at 28–32 days and pregnancy loss included farm, pre-synchronization (presynch or no presynch), semen (conventional or sexed), season (summer, fall and winter), type of insemination (TAI or AIE), age (month) of heifer, treatment (CIDR-4 vs. CIDR-5) and two-way interactions with treatment. Statistical model for rates of TAI and AIE consisted of pre-synchronization (presynch or no presynch), season (summer, fall and winter), age (month) of heifer, treatment (CIDR-5 vs. CIDR-4) and two-way interactions with treatment. Results of logistic regression-stepwise elimination procedure were reported as odds ratio along with 95% confidence interval with significance level *p* < 0.05 for selected variables.

## 3. Results

**Figure 2:**
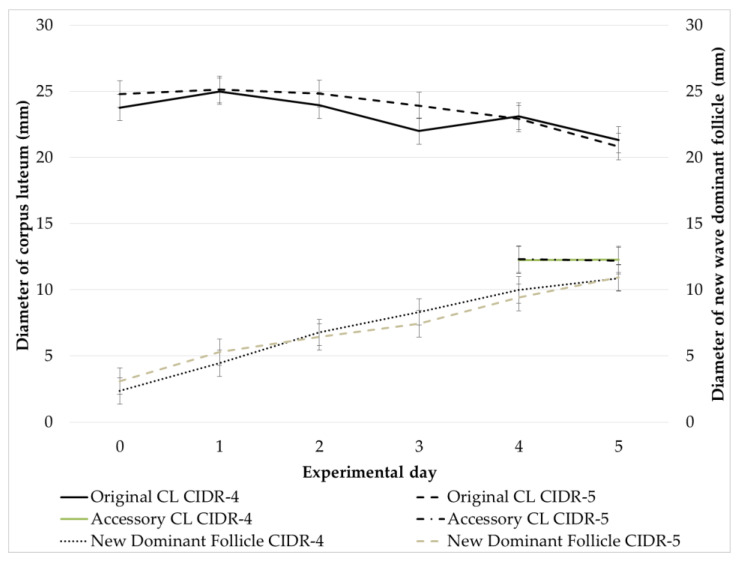
Development of original CL, accessory CL and new wave dominant follicle during experimental period.

In subset of the experiment, ovulation rate and accessory CL formation following initial GnRH injection did not differ between CIDR-4 (77.8%, 7/9; 55.6%, 5/9) and CIDR-5 (60%, 9/15; 46.7%, 7/15) groups, respectively. Similarly, there was no difference for the rate of new dominant follicle development between CIDR-4 (100%, 9/9) and CIDR-5 (93.3%, 14/15) groups. Moreover, diameters of original CL and accessory CL along with new wave dominant follicle did not differ between CIDR-4 and CIDR-5 groups (Figure 2).

**Figure 3:**
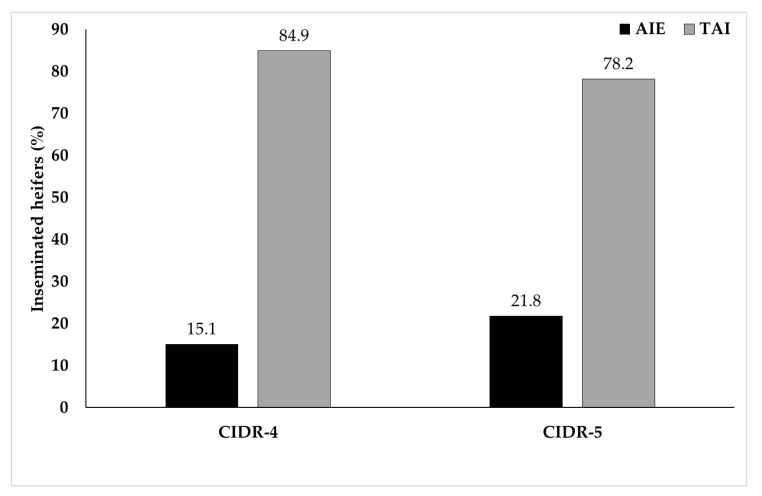
The distribution of AI at detected estruses (AIE, black bars) and timed artificial insemination (TAI, gray bars) in CIDR-4 and CIDR-5 groups. Rate of AIE was tended (*p* < 0.09) to be higher in CIDR-5 group than that in CIDR-4 group.

Separate Chi-square analysis indicated that rate of AIE was tended (Figure 3; *p* < 0.09) to be higher in CIDR-5 group (21.8% [45/206]) than that in CIDR-4 group (15.1% [29/192]). Whereas, rate of TAI was lower in CIDR-5 (78.2% [161/206]) group than that in CIDR-4 group (84.9% [163/192].

P/AI on days 28–32 and 45–60 after insemination did not differ between CIDR-4 (44.3%, [85/192]; 43.8%, [84/192]) and CIDR-5 (51.9%, [107/206]; 51.0, [105/206]) groups, respectively. Moreover, there were three pregnancy losses between two pregnancy diagnosis, and pregnancy losses did not differ between CIDR-4 (1.2%, 1/85) and CIDR-5 (1.9%, 2/107) groups. Logistic regression analysis stepwise procedure revealed significant effects of sexed semen and interaction effects of treatment by age of heifer and treatment by presynchronization on P/AI at 28–32 days after insemination.

Overall, the chance for P/AI was 2.48 (0.99–6.20) times higher (*p* < 0.05) in heifers inseminated with conventional semen (50.6%, [172/340]) compared to those inseminated with sexed semen (34.5%, [20/58) at 28–32 days after insemination. However, separate Chi-square analysis indicated that there was a significant interaction (*p* < 0.01) between semen type (conventional vs. sexed) and insemination type (AIE vs. TAI) for P/AI regardless of treatment (Table 1). In this matter, P/AI was significantly (*p* < 0.01) higher in heifers inseminated with conventional semen (65.9% [27/41]) than those inseminated with sexed semen (24.2% [8/33]) following AIE (Table 1). Whereas, P/AI was similar between heifers inseminated with conventional semen (48.5% [145/299]) and those inseminated with sexed semen (48% [12/25]) following TAI (Table 1).

**Figure 4:**
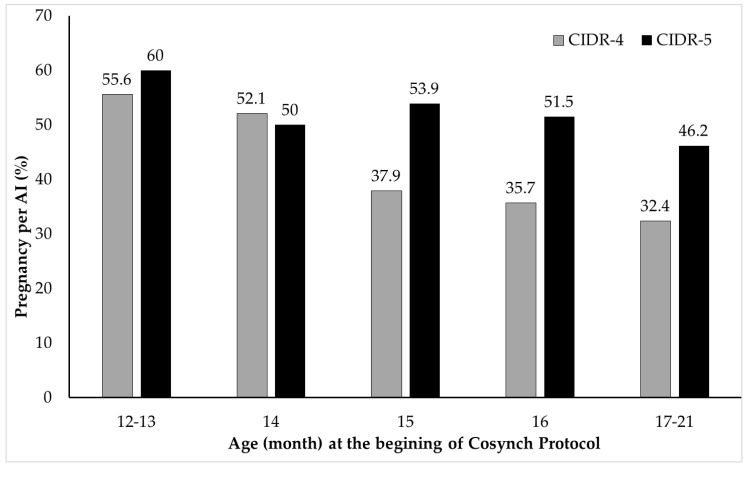
Significant (*p* < 0.01) interaction effect of treatment (CIDR-4 vs. CIDR-5) by age of heifer indicates lower pregnancy per AI in CIDR-4 (gray bars) group as heifers age increased compared to CIDR-5 (black bars) group.

**Figure 5:**
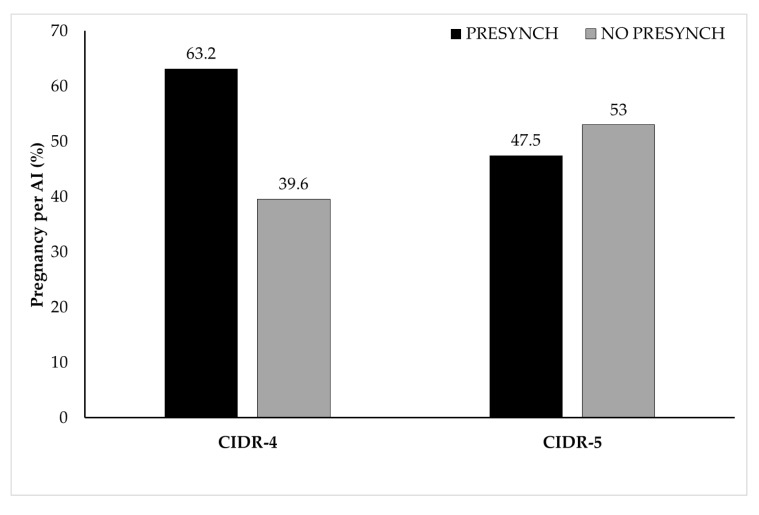
Significant (*p* < 0.05) interaction effect of treatment (CIDR-4 vs. CIDR-5) by presynchronization indicates lower pregnancy per AI in heifers without presynchronization (gray bars) compared to those with presynchronization (black bars) in CIDR-4 group. While pregnancy per AI in heifers with or without presynchronization were similar in CIDR-5 group.

With regard to a significant (*p* < 0.01) interaction effect of treatment by age of heifer, the chance for P/AI was 0.95 (0.91–0.99) times more declined as heifers’ age (12–13, 14, 15, 16 and 17–21 months) proceeded in CIDR-4 group (55.6%, [15/27]; 52.1%, [37/71]; 37.9%, [11/29]; 35.7%, [10/28]; 32.4%, [12/37]) compared to those in CIDR-5 group (60.0%, [15/25]; 50.0%, [35/70], 53.9%, [28/52]; 51.5%, [17/33], 46.2%, [12/26]); respectively, at 28–32 days after insemination (Figure 4). Moreover, a significant (*p* < 0.05) interaction effect (odds ratio estimate: 0.32 [0.12–0.90]) of treatment by presynchronization indicates that the chance of P/AI was higher in pre-synchronized heifers (63.2%, [24/38]) compared to that in those without presynchronization (39.6%, [61/154]) in CIDR-4 group at 28–32 days after insemination. Whereas, the chance of P/AI was similar between pre-synchronized (47.5%, [19/40]) and not pre-synchronized (53.0%, [88/166) heifers in CIDR-5 group at 28–32 days after insemination (Figure 5).

After CIDR removal, there was no difference for rates of AIE between CIDR-4 (15.1%, [29/192]) and CIDR-5 (21.8%, [45/206]) groups. Logistic regression analysis stepwise procedure indicated significant effects of age of heifer and presynchronization on rates of AIE. In this matter, rates of AIE were 6.88 (2.20–21.47) times higher (*p* < 0.01) in heifers 12–13 months old (28.9%, [15/52]]) and 4.49 (1.64–12.34) times higher (*p* < 0.01) in heifers 14 months old (24.8%, [35/141]) compared to those 17–21 months old (7.9%, [5/63]). Whereas, rates of AIE in heifers 15 (11.1%, [9/81]) and 16 (16.4%, [10/61]) months of age did not differ from heifers 17–21 months old (Table 1). Furthermore, rates of AIE were 4.51 (1.75–11.60) times higher (*p* < 0.01) in heifers without presynchronization (21.3%, [68/320]) compared to those with presynchronization (7.7%, [6/78]).

## 4. Discussion

No differences were found for accessory CL formation following four versus five days CIDR insertion during 5-day CIDR-Cosynch protocol in dairy heifers in this study. This current finding was not in agreement with a previous study in which feeding synthetic progesterone (melengestrole acetate, [MGA]) concomitant with GnRH injection disrupted formation of CL at the beginning of Ovsynch protocol conducted at early luteal phase in Holstein dairy heifers [15]. This discrepancy between current and previous studies could be due to differences in potential of these two progesterone content, sources and doses. In this matter, MGA is active syntetic progestin. Whereas, there is a natural progesterone molecule in CIDR. Moreover, MGA was given orally, and CIDR was inserted intravaginally. In addition, MGA was given 0.5 mg/day/head; whereas, approximately 100 mg P4 is released daily from CIDR through vagina. Perhaps, MGA could be more potent due to its content, oral route use and its dosage in comparison to CIDR leading to disruption of accessory CL formation. Although both ovulation rate following initial GnRH administration and presence of a new CL at PGF2α injection were higher in dairy heifers treated with 5-day CIDR-Cosynch protocol compared to those treated with no GnRH administration at the beginning of modified 5-day CIDR-Cosynch protocol, P/AI was not different between treatments [16].

Similar to Lima et al. [16], no main effect of concomitant use of GnRH and P4 on P/AI in present study could indicate that follicle turnover following initial GnRH during 5-day Cosynch protocol could not be a limiting factor for fertility. This could be due to faster follicular wave development in dairy heifers because new dominant follicles were developed despite no ovulation following initial GnRH administration in current study. In this regard, it has been reported that dairy heifers had more follicular waves than cows despite similar interovulatory intervals [6]. Furthermore, no increase in P/AI following modification of CIDR administration during Cosynch protocol in present study could also be due to faster follicular wave development regardless of concomitant use of GnRH at the time of CIDR insertion in dairy heifers, since no beneficial effect of initial GnRH administration at the time of CIDR insertion on P/AI during 5-day CIDR-Cosynch protocol in dairy heifers was reported [17]. Lima et al. [8] attributed lower ovulation rate following initial GnRH injection during CIDR-Cosynch protocol to suppression of LH release due to elevated P4 concentration. However, ovulation rate following initial GnRH injection at favorable stage of the estrous cycle was not statistically higher in CIDR-4 group than that in CIDR-5 group despite one day delay of P4 supplementation to eliminate P4 pressure on LH release during CIDR-Cosynch protocol in this study. This discrepancy between current study and previous studies could be related to different GnRH compounds and dose. In this regard, Giordano et al., [18] reported that lactating dairy cows had lower LH surge under high P4 environment, and 200 μg of GnRH increased LH pike compared to regular dose of 100 μg of GnRH regardless of P4 status during Double-Ovsynch protocol.

Higher overall P/AI after the first AI in dairy heifers inseminated with conventional semen compared to those inseminated with sexed semen in present study was in agreement with Chebel et al. [19]. Similarly, Oikawa et al. [20] reported that almost 80% of conception rate with conventional semen was achieved with sexed semen following the first AI in Holstein heifers. Moreover, no interaction effect between treatment and semen type could indicate that lower P/AI with use of sexed semen in both CIDR-4 and CIDR-5 groups could be solely due to sexed semen itself rather than length of CIDR treatment in Cosynch protocol in current study. In this matter, Silva et al. [21] reported that P/AI was higher following TAI in 5-day CIDR Cosynch protocol than that in following AIE at the first insemination with use of sex-sorted semen compared to conventional semen in dairy heifers; while, overall P/AI was lower in sex-sorted semen. Likewise, similar P/AI following TAI with conventional and sexed semen could reveal efficacy of TAI with use of sexed semen and efficacy of AIE with conventional semen in 5-day CIDR Cosynch protocol in dairy heifers; while, overall P/AI was lower in sex-sorted semen in present study.

Age of heifers at puberty is a critical production trait to ensure good lifetime productivity in dairy heifers [22]. In dairy herds, the puberty should occur by 12 months of age and the main influences on the timing of puberty are body weight and age [22,23]. In present study, heifers not younger than 365 days of age were assigned randomly in two groups to receive a 4- or 5-day CIDR insertion in 5-day-Cosynch protocol. Although P/AI at the first breeding was numerically maximal in heifers at an age of 12–13 months, and P/AI gradually decreased as heifers age at first breeding increased, there was no main effect of age on P/AI in present study. However, a significant interaction effect of treatment by age of heifer on P/AI indicated more declining trend in P/AI as heifers’ age proceeded in CIDR-4 group compared to those in CIDR-5 group. In this matter, higher P/AI in heifers inseminated at 15 months of age and older in CIDR-5 group compared to those in CIDR-4 group could reveal benefit of one day longer CIDR exposure among older heifers probably being acyclic in current study. One other explanation of low P/AI in older heifers of CIDR-4 group could also be smaller follicular diameter throughout the study period. Fishman-Holland et al. [12] showed that dairy heifers treated with 4-day CIDR Cosynch protocol require more time to complete follicular development after CIDR removal, resulting in longer time interval from CIDR removal to ovulation compared with heifers which treated with 5-day CIDR-Cosynch protocol. In current study, it is more probably that one-day delaying CIDR insertion after initial GnRH injection could increase the chance for growth of smaller follicles in heifers with proper growth rather than late growing heifers.

Because aged heifers were not inseminated due to inadequate growth and/or not being detected in estrus until to be enrolled to this experiment, incidence of acyclic heifers could be higher in aged heifers. Similar to current data, Brickell et al. [24] reported that maximal conception rate was achieved in younger dairy heifers at first insemination. Likewise, Say et al. [25], reported that P/AI was higher in dairy heifers 15–16 months old than those 17–18 months old in 5 day Cosynch protocol. Higher P/AI at the first AI in heifers at an age of 12–13 months in current study could be better growth and development during the rearing period in current study and study conducted by Brickell et al. [24]. In contrast, Kuhn et al. [26] reported that conception rates were maximal for inseminations at 15 to 16 months of age, and conception rates were lower for inseminations at less than 15 months and more than 16 months of age in dairy heifers. Regarding lower fertility in older heifers at the first AI, current study was in agreement with Kuhn et al. [26].

Masello et al. [27] reported similar the first service P/AI following TAI in 5-day CIDR-Cosynch protocol or combinations of AIE and TAI in 5-day CIDR-Cosynch protocol initiated nine days after presynchronization with two or three PGF2α administrations fourteen days apart in dairy heifers. Similarly, there was no main effect of presynchronization prior to both CIDR-4 and CIDR-5 groups in 5-day CIDR-Cosynch protocol in heifers although heifers were not allowed to be inseminated during presynchronization in current study. Significant interaction effect of treatment by presynchronization indicates that P/AI was higher in pre-synchronized heifers compared to that in those without presynchronization in CIDR-4 group. In CIDR-4 group, higher P/AI following presynchronization could be attributed to start Cosynch protocol at early diestrus leading to ovulation or turnover of dominant follicle and new follicular wave development without excessive P4 suppression to compromise LH receptors by delaying CIDR insertion. Regarding this hypothesis, P/AI was numerically lower in heifers following presynchronization in CIDR-5 group than that in CIDR-4 group due probably to suppression of ovulation or turnover of dominant follicle. Alternatively, function of original CL could be compromised with concomitant use of CIDR and GnRH. Because all heifers were presynchronized before initiation of Cosynch protocol in subset of heifers, no differences between CIDR-4 and CIDR-5 group following presynchronization for follicle turnover, ovulation and development of original and accessory CL along with dominant follicle could indicate that lower P/AI in CIDR-5 group could be due to less functional CL development following P4 supplementation concurrent with GnRH administration at the beginning of Cosynch protocol. Since P4 concentration were not determined in this study, it warrants further study. In heifers without presynchronization, lower P/AI could be owing to asynchronized follicle development at the beginning of Cosynch protocol leading to ovulation of aged follicle in CIDR-4 group than that in CIDR-5 group in which asynchronized dominant follicle development could be slowed down with immediate CIDR insertion. Likewise, Oosthuizen et al. [28] reported increase in P/AI following presynchronization with PGF2α seven days prior to 7 days Cosynch CIDR protocol involving TAI with sex-sorted semen 72 h after from CIDR removal in beef heifers. In contrast, similar P/AI between pre-synchronized and not pre-synchronized heifers could indicate no beneficial effect of presynchronization in CIDR-5 group. Therefore, significant interaction effect could reveal beneficial effect of presynchronization on CIDR-4 protocol as a reproductive management of choice, and it warrants further study.

In present study, no difference for rates of AIE prior to TAI between CIDR-4 and CIDR-5 groups could reveal that both groups equally effective for synchronization of ovulation in 5-day Cosynch protocol. Likewise, no difference for the rate of estrus at TAI in dairy heifers with or without initial GnRH administration in 5-day Cosynch protocol [16]. No effect of initial GnRH administration in 5-day Cosynch protocol on premature estrus in present study, and on the rate of estrus at TAI in a study conducted by Lima et al. [16] could indicate that inadequate response to initial GnRH could not disrupt synchronization of ovulation via spontaneous development of new follicular wave in dairy heifers. In current study, significant effects of age of heifer on rates of AI following estrous detection prior to TAI indicated that incidence of premature estrus prior to TAI increased as heifers age proceeded. This trend in premature estruses could be due to short cycles in younger heifers with first pubertal estruses. Moreover, significantly higher rate of AI following estrous detection prior to TAI in heifers without presynchronization than that in those with presynchronization could indicate efficacy of presynchronization both in CIDR-4 and CIDR-5 groups in 5-day Cosynch protocol.

## 5. Conclusions

In conclusion, there was no effect of concomitant administrations of GnRH and intravaginal P4 at the beginning of 5-day CIDR Cosynch protocol on P/AI along with the rate of AI following estrous detection prior to TAI in dairy heifers. However, higher P/AI following presynchronization in CIDR-4 group could be considered for reproductive management in dairy heifers and it warrants further research. Moreover, higher P/AI in CIDR-5 group in aged dairy heifers could reveal the necessity of longer P4 administration in Cosynch protocol for better fertility. Furthermore, sexed semen could be preferred in TAI and conventional semen could be preferred for AI at detected estrus in 5-day CIDR Cosynch protocol and it needs further large field experiments.

## Figures and Tables

**Table 1:** Distribution of P/AI (% and *n*/*n*) on days 28–32 after insemination for treatment, use of presynchronization or not, use of semen type* (sexed [SS] or conventional [CS] semen), insemination time (AIE or TAI) among age of heifers.

	CIDR4						CIDR5					
**Age of heifer** **(month)**	All Ages	12–13	14	15	16	17–21	All ages	12–13	14	15	16	17–21
**Presynch** **+CS+TAI**	62.9 22/35	80 4/5	60 15/25	100 3/3	0 0/1	0 0/1	48.7 18/37	66.7 2/3	50 7/14	57.1 8/14	20 1/5	0 0/1
**Presynch** **+CS+AIE**	66.7 2/3		100 1/1		50 1/2		33.3 1/3			0 0/1	50 1/2	
**No Presynch +SS+TAI**	23.1 3/13	0 0/2	30 3/10			0 0/1	75 9/12	100 2/2	77.8 7/9			0 0/1
**No Presynch +SS+AIE**	28.6 2/7	25 1/4			100 1/1	0 0/2	23.1 6/26	16.7 1/6	20 2/10	28.6 2/7	100 1/1	0 0/2
**No Presynch** **+CS+TAI**	38.3 44/115	60 9/15	47.6 10/21	28 7/25	27.3 6/22	37.5 12/32	54.5 61/112	60 6/10	48.2 13/27	60 18/30	52.2 12/23	54.6 12/22
**No Presynch** **+CS+AIE**	63.2 12/19	100 1/1	57.1 8/14	100 1/1	100 2/2	0 0/1	75 12/16	100 4/4	60 6/10		100 2/2	
**Total**	44.3 85/192	55.6 15/27	52.1 37/71	37.9 11/29	35.7 10/28	32.4 12/37	51.9 107/206	60 15/25	50 35/70	53.9 28/52	51.5 17/33	46.2 12/26

**SS**: Sexed semen; **CS**: Conventional semen; **TAI**: Timed Artificial Insemination, **AIE**: AI on detected estrus. * In presynchronized heifers, sexed semen was not used.

## Data Availability

The data presented in this study are available on request from the corresponding author.
